# Oxidative stress and carbon metabolism influence *Aspergillus flavus* transcriptome composition and secondary metabolite production

**DOI:** 10.1038/srep38747

**Published:** 2016-12-12

**Authors:** Jake C. Fountain, Prasad Bajaj, Manish Pandey, Spurthi N. Nayak, Liming Yang, Vinay Kumar, Ashwin S. Jayale, Anu Chitikineni, Weijian Zhuang, Brian T. Scully, R. Dewey Lee, Robert C. Kemerait, Rajeev K. Varshney, Baozhu Guo

**Affiliations:** 1 Department of Plant Pathology, University of Georgia, Tifton, GA, USA; 2 USDA-ARS Crop Protection and Management Research Unit, Tifton, GA, USA; 3 International Crop Research Institute for the Semi-Arid Tropics (ICRISAT), Hyderabad, Telangana, India; 4 Fujian Agricultural and Forestry University, Fuzhou, Fujian, China; 5 USDA-ARS US Horticultural Research Laboratory, Fort Pierce, FL, USA; 6 Department of Crop and Soil Sciences, University of Georgia, Tifton, GA, USA

## Abstract

Contamination of crops with aflatoxin is a serious global threat to food safety. Aflatoxin production by *Aspergillus flavus* is exacerbated by drought stress in the field and by oxidative stress *in vitro.* We examined transcriptomes of three toxigenic and three atoxigenic isolates of *A. flavus* in aflatoxin conducive and non-conducive media with varying levels of H_2_O_2_ to investigate the relationship of secondary metabolite production, carbon source, and oxidative stress. We found that toxigenic and atoxigenic isolates employ distinct mechanisms to remediate oxidative damage, and that carbon source affected the isolates’ expression profiles. Iron metabolism, monooxygenases, and secondary metabolism appeared to participate in isolate oxidative responses. The results suggest that aflatoxin and aflatrem biosynthesis may remediate oxidative stress by consuming excess oxygen and that kojic acid production may limit iron-mediated, non-enzymatic generation of reactive oxygen species. Together, secondary metabolite production may enhance *A. flavus* stress tolerance, and may be reduced by enhancing host plant tissue antioxidant capacity though genetic improvement by breeding selection.

Maize and peanut are staple food crops throughout the world with 1.02 billion and 45.65 million metric tons of production, respectively, in 2013^1^. These crops are particularly important in developing countries in Africa and Asia where aflatoxin contamination is most severe^2^,^3^. Contamination of maize and peanut with aflatoxins, the carcinogenic secondary metabolites of *Aspergillus flavus*, poses a global threat to food safety and security. Long term exposure to aflatoxin through contaminated foodstuffs has been associated with a myriad of hepatic diseases including cirrhosis, hepatitis, and hepatocellular carcinoma along with birth defects, immune deficiencies, and acute toxicity^4^,^5^.

Given the importance of aflatoxin contamination remediation, extensive research has been performed since the 1970’s to enhance host plant resistance^6^,^7^,^8^. However, a lack of diverse, resistant germplasm for aflatoxin contamination along with a high degree of environmental and genotype × environmental interaction influences on resistance has posed difficulties in breeding programs^9^,^10^,^11^. Environmental stresses including drought and heat stress can exacerbate aflatoxin contamination in maize and peanut^12^,^13^. This increase in aflatoxin contamination under drought stress in particular has motivated numerous research efforts endeavoring to address why *A. flavus* and other Aspergilli produce aflatoxin in stressed environments.

Over the last decade, several studies have elucidated the biosynthetic pathway for aflatoxin production and have begun to characterize upstream factors regulating its expression^14^,^15^,^16^. Aflatoxin biosynthesis is carried out by a 25 gene-cluster that is highly conserved among toxigenic isolates of *A. flavus*^16^. The production of aflatoxin has been shown to be regulated through several means, although the practical role of aflatoxin and various other secondary metabolites in *A. flavus* biology and/or its interactions with other organisms including plant host-pathogen interactions is still not clear. Reactive oxygen species (ROS) and their reactive products including peroxidized lipids have been shown to induce and to be required for aflatoxin production^17^. Aflatoxin production has also been demonstrated to be under the regulation of oxidative stress-responsive signaling mediated by transcription factors such as AtfB, AP-1, and VeA^17^,^18^,^19^,^20^,^21^. Given that drought stress results in the accumulation of ROS and peroxidized lipids in host plant tissues, it is hypothesized that these compounds may exacerbate the production of aflatoxin during fungal colonization of stressed host tissues^22^,^23^,^24^,^25^. This correlation between ROS and aflatoxin production has also led to the hypothesis that aflatoxin production may function in the remediation of oxidative stress^8^,^17^,^21^,^26^,^27^,^28^.

In addition to ROS, carbohydrate availability and metabolism have also been shown to influence aflatoxin production. Earlier studies by Davis and Diener^29^ demonstrated the requirement of hexose sugars for the production of aflatoxins *in vitro* with compounds occurring later in carbohydrate metabolism (e.g. the tricarboxylic acid, TCA, cycle) being unable to support aflatoxin production. The close association of a simple sugar metabolic gene cluster with the aflatoxin biosynthetic gene cluster has also been demonstrated in *A. parasiticus*, though a similar association was not observed in all *Aspergillus spp*^16^,^30^. In *A. flavus* isolates, supplementing culture media with alternative carbon sources such as peptone results in reduced or inhibited aflatoxin production^31^,^32^. The modulation of aflatoxin production through cultural methods including carbon source variation and ROS amendment has shown that isolates that produce aflatoxin tend to exhibit greater tolerance to oxidative stress, and that the reduction of aflatoxin production can adversely affect said tolerance^18^,^28^.

In order to better understand the regulation of aflatoxin production in response to carbohydrate availability and oxidative stress, and to investigate the possible role of aflatoxin production in stress responses, a global gene expression approach is needed. In this study, we examined the transcriptomes of toxigenic and atoxigenic isolates of *A. flavus* and their responses to increasing levels of oxidative stress and altered carbon source availability in culture medium. Understanding the molecular mechanisms linking stress responsive signaling and aflatoxin production pathways could provide insights into the practical role of aflatoxin production and considerations for enhancing host resistance for aflatoxin contamination.

## Results

### Transcriptome sequencing

To examine the transcriptomic responses of toxigenic and atoxigenic isolates of *A. flavus* to increasing levels of oxidative stress in aflatoxin production conducive and non-conducive media, we cultured three toxigenic (AF13, NRRL3357, and Tox4) and three atoxigenic (AF36, Aflaguard, and K54A) in either YES or YEP medium amended with different concentrations of H_2_O_2_ for RNA sequencing (Supplementary Table 1). In total, ~4.7 billion reads were generated from 64 libraries with an average of 73 million reads per library. Two of the 64 libraries sequenced contained low read counts and were excluded from the analysis. Following quality check, an average of 9 to 10% of the reads were removed, which were further filtered for rRNA contamination prior to mapping. Following quality filtration, 2.5 billion quality filtered reads with an average of 40.3 million reads per sample were obtained of which 92.27% were mapped to the *A. flavus* genome. The raw and processed RNA sequencing data can be accessed at http://ceg.icrisat.org/gt-bt/alfavus_1/home.asp.

**Figure 1:**
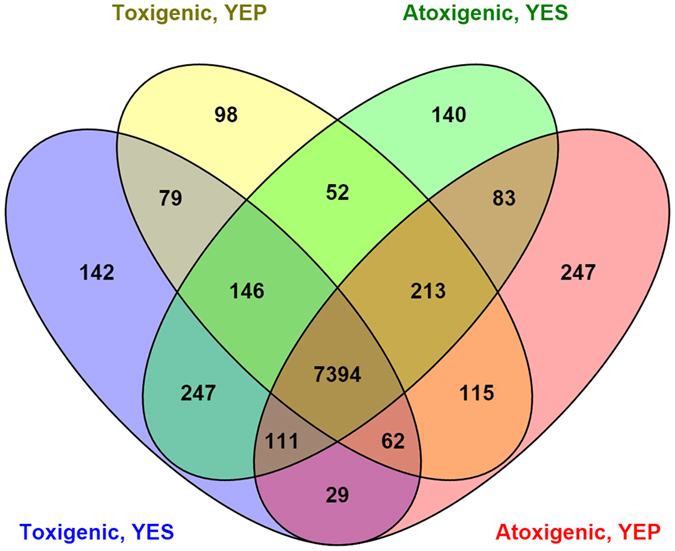
Venn diagram of genes expressed in toxigenic and atoxigenic isolates in YES (yeast extract sucrose) and YEP (yeast extract peptone) media. Genes with FPKM ≥ 2 were considered to be expressed. Those genes expressed exclusively in YES (aflatoxin conducive) or YEP (aflatoxin non-conducive) medium in toxigenic (Tox4, AF13, and NRRL3357) and atoxigenic (Aflaguard, AF36, K54A) isolates were identified using Venny 2.1.0.

Of the 13,487 genes present in the *A. flavus* NRRL3357 genome, the gene expression data analysis showed that a total of 11,144 genes (82.63% of the reference) were identified in the dataset (Supplementary Data 1). Of those detected, 9,158 genes (82.18%) were found to possess an FPKM ≥ 2 in at least one isolate-treatment combination. Among these expressed genes, 319 genes (3.48%) and 470 genes (5.13%) were expressed uniquely in the toxigenic and atoxigenic isolates, respectively. Also, 529 genes (5.78%) were expressed only in the conducive YES medium while 460 genes (5.02%) were expressed only in the non-conducive YEP medium (Fig. 1, Supplementary Data 2). In total, 7,394 genes (80.74%) were expressed in both toxigenic and atoxigenic isolates when cultured in either medium (Fig. 1, Supplementary Data 2).

### Toxigenic isolate responses to oxidative stress are related to their ability to produce aflatoxin

On examining the level of oxidative tolerance of these isolates, our previous studies found that isolates with greater levels of aflatoxin production tend to tolerate greater levels of oxidative stress^32^. In order to examine the transcriptional responses of the toxigenic isolates to oxidative stress we identified the significantly differentially expressed genes in the isolates in YES medium in response to increasing levels of oxidative stress. Toxigenic isolates which produce greater levels of aflatoxin were found to exhibit fewer differentially expressed genes in response to stress than those which produced less aflatoxin (Table 1). For the transcripts observed to be differentially expressed in the toxigenic isolates, those involved in membrane transport, carbon metabolism, stress signaling, antioxidant mechanisms, and secondary metabolite production were enriched (Supplementary Data 3). Comparing high and moderate aflatoxin producing isolate GO enrichments also indicates that the high toxin producing isolates, Tox4 and AF13, exhibit similar responses at higher levels of stress as those employed by the moderate producing NRRL3357 isolate under less stress (Supplementary Data 3).

**Figure 2:**
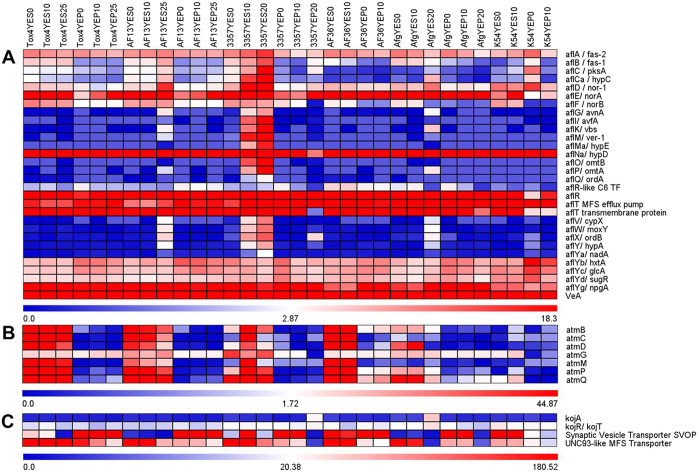
Heatmap of secondary metabolite biosynthetic gene expression. Expression of genes involved in the biosynthesis of aflatoxin (**A**), aflatrem (**B**), and kojic acid (**C**) are plotted with colors corresponding to the FPKM’s of the genes within each isolate and treatment combination. For each heatmap, individual keys are located at the base of each plot with FPKM values increasing from blue to red. Isolate and treatment labels at the top of the figure apply throughout.

As expected, transcripts encoding components of the aflatoxin biosynthetic pathway were found to be up-regulated in response to increasing stress. These included *nor-1, verB, ver-1, omtA*, and *omtB* along with more cryptic members of the aflatoxin biosynthetic gene cluster whose role(s) in aflatoxin production have yet to be identified including *aflS* and *aflV* (Fig. 2A)^16^. In addition to these, transcripts encoding the production of additional secondary metabolites were differentially expressed in response to increasing stress. Transcripts encoding members of the isoprenoid biosynthetic pathway, which forms the basis for the production of the tremorogenic mycotoxin aflatrem^33^, were also found to be up-regulated in the isolates in a similar manner observed for aflatoxin biosynthetic genes. The aflatrem biosynthetic components *atmB, atmC, atmD, atmG, atmM, atmP*, and *atmQ*^34^ were expressed in all the toxigenic isolates in YES medium (Fig. 2B). The *atmG* gene, encoding a putative geranylgeranyl pyrophosphate synthetase (GGPS), was consistently expressed in both the toxigenic and atoxigenic isolates regardless of H_2_O_2_ concentration or culture medium (Fig. 2B). Also, a synaptic vesicle transporter SVOP transcript, which has been shown to be involved in kojic acid biosynthesis^35^, was found to be upregulated in NRRL3357 at 10 mM H_2_O_2_ in YES while it was down-regulated in the more virulent Tox4 and AF13 isolates at 25 mM H_2_O_2_ (Fig. 2C). Other kojic acid biosynthetic genes including *kojA, kojR*, and *kojT*^35^ were stably expressed in all the toxigenic isolates regardless of treatment with H_2_O_2_ or culture medium (Fig. 2C). Numerical FPKM data for that appearing in Fig. 2 can be found in Supplementary Table 2.

### Toxigenic and atoxigenic isolates exhibit similar yet distinct responses to oxidative stress

In comparison to the toxigenic isolates, the atoxigenic isolates exhibited distinct responses to oxidative stress, though there were some similarities. Among the atoxigenic isolates, those which are used as biological controls, Aflaguard and AF36, were previously found to tolerate higher levels of oxidative stress than non-adapted isolates including K54A^32^. In the present study, these biological control isolates exhibited fewer DEGs in comparison to K54A (Table 1). In addition, the atoxigenic isolates, which generally tolerated lower levels of oxidative stress than the toxigenic isolates in the previous study^32^, tended to exhibit greater numbers of significant DEGs than the toxigenic isolates, particularly at higher levels of stress (Table 1).

As in the toxigenic isolates, transcripts observed to be differentially expressed in the atoxigenic isolates included those involved in membrane transport, carbon metabolism, stress signaling, and antioxidant mechanisms (Supplementary Data 3). As expected, transcripts encoding components of the aflatoxin biosynthetic pathway were either not significantly regulated, or not expressed in the atoxigenic isolates (Fig. 2A) depending on the mutation in the aflatoxin gene cluster present in each isolate^36^,^37^. Aflatrem biosynthetic genes were also found to be expressed in Aflaguard and AF36, but not in K54A while kojic acid biosynthesis genes were expressed in all of the atoxigenic isolates in YES medium (Fig. 2).

Transcripts encoding chitin metabolic processes were down-regulated in response to increasing stress in the toxigenic isolates including those for chitinase and chitosanase (Supplementary Data 3). These same transcripts tended to be up-regulated in the atoxigenic isolates (Supplementary Data 3). Also, transcripts encoding antioxidant enzymes including thioredoxin peroxidase and thioredoxin reductase also tended to be upregulated in response to increasing stress in both the toxigenic and atoxigenic isolates (Supplementary Data 3). Cytochrome p450 monooxygenase genes, in addition to those expressed in the aflatoxin and aflatrem biosynthetic pathways, were also upregulated in both the toxigenic and atoxigenic isolates in response to increasing stress (Supplementary Data 3). Transcripts encoding products involved in aminobenzoate degradation were shown to be generally up-regulated in the toxigenic and atoxigenic isolates under increasing levels of stress (Supplementary Data 3). An exception to this was Tox4 which showed a significant down-regulation in an amidohydrolase family protein-encoding transcript (AFLA_076800) (Supplementary Data 3). Also, transcripts encoding antibiosis and drug resistance proteins such as major facilitator superfamily (MFS) transporters and multidrug resistance proteins were up-regulated in the toxigenic isolates with increasing stress (Supplementary Data 3 and 4). In the atoxigenic isolates, transcripts encoding products capable of functioning in both antibiotic biosynthesis and additional pathways such as carbon metabolism and proteolysis were differentially expressed in response to increasing stress.

### Medium carbon source affects secondary metabolite gene expression and production

**Figure 3:**
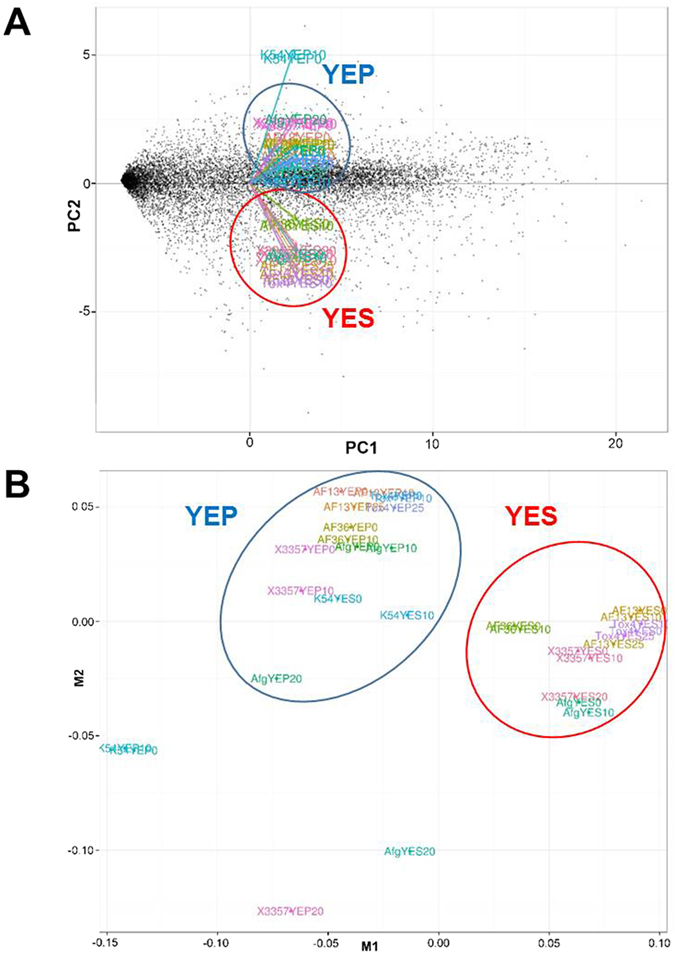
Principal component and multi-spatial analyses of *Aspergillus flavus* isolate expression profiles. Principle component (**A**) and multi-spatial (**B**) analyses of the expression profiles of the isolates in both YES (yeast extract sucrose) and YEP (yeast extract peptone) media with increasing levels of oxidative stress. The patterns present in the data indicate a clear segregation of the isolate profiles based mainly on culture medium carbon source.

**Figure 4:**
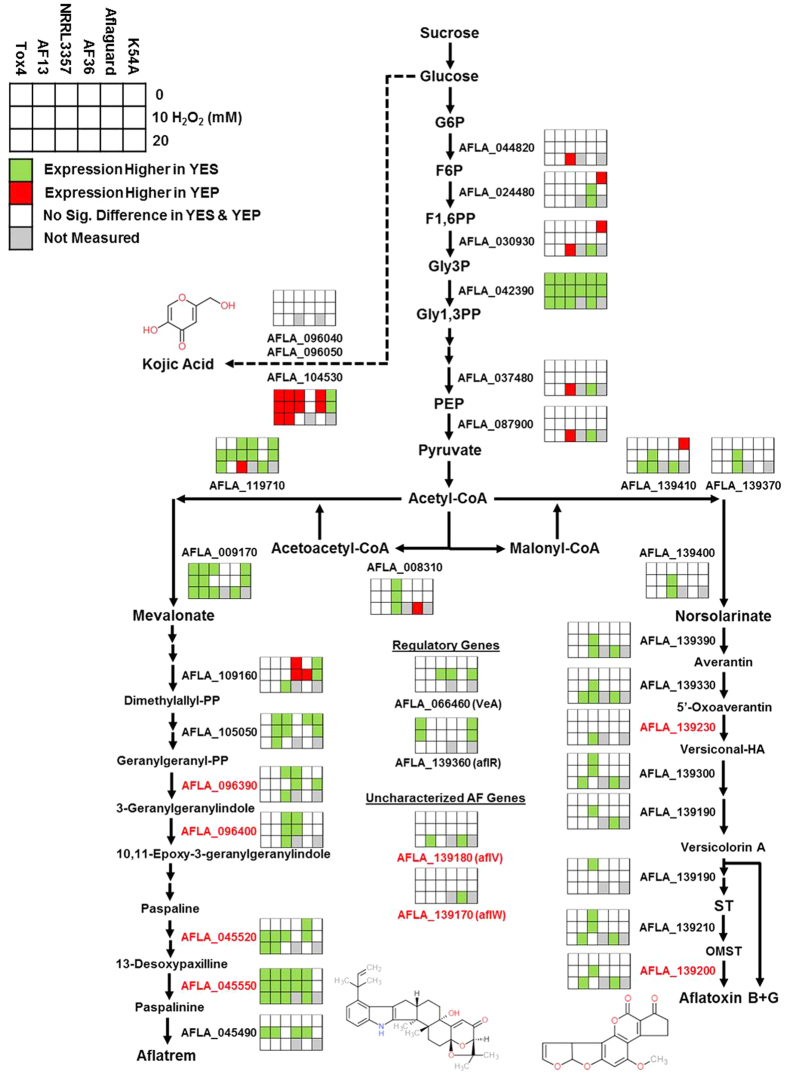
Differential expression of genes involved in glycolysis and secondary metabolite biosynthesis. The 6 × 3 tables beside the gene annotations indicate significant changes in gene expression between YES (yeast extract sucrose) and YEP (yeast extract peptone) media for each isolate when exposed to H_2_O_2_ (0, 10, and 20 or 25 mM). Green and red colors indicate significantly higher expression of the genes in YES and YEP, respectively. White color represents no significant difference between YES and YEP; and gray color represents comparisons not measured in the experiment. Red colored points in molecular models represent the occurrence of oxygen atoms. Red colored text for gene annotations represent cytochrome p450 monooxygenase-coding genes present in the aflatoxin and aflatrem biosynthetic pathways. Abbreviations: G6P, glucose-6-phosphate; F6P, fructose-6-phosphate; F1,6 P, fructose-1,6-bisphosphate; Gly3P, glyceraldehyde-3-phosphate; Gly1,3PP, glyceraldehyde-1,3-bisphosphate; PEP, phosphoenolpyruvate.

**Figure 5:**
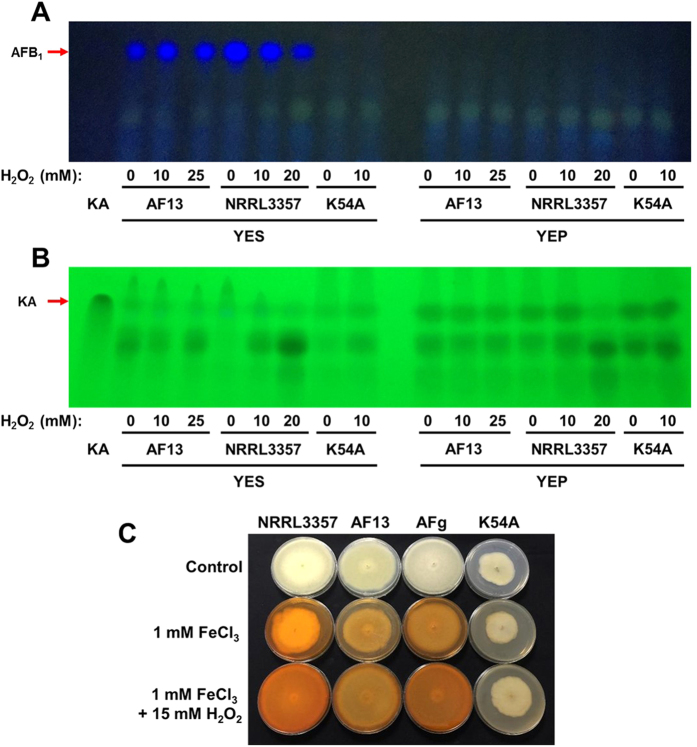
Aflatoxin and kojic acid production assays. Thin layer chromatography of liquid culture medium from the cultured isolates was performed for aflatoxin B1 (**A**) and kojic acid (**B**) with UV excitation at 365 and 254 nm, respectively. Aflatoxin was produced by the toxigenic isolates only in YES (yeast extract sucrose) while no aflatoxin production was observed for K54A in either medium. Kojic acid was shown to be produced by all isolates at a higher level in YEP (yeast extract peptone) than in YES (**B**), and seemed to be stimulated by the addition of H_2_O_2_ (**C**). In the solid medium assay based on Bentley^66^ (**C**), the low production of kojic acid by K54A in the solid culture assay may be due to compositional differences in PDA and YES. Images are representative of three experimental replicates.

Culturing the toxigenic and atoxigenic isolates in media with different carbon sources, sucrose or peptone, significantly altered the expression profiles of the isolates in response to increased levels of oxidative stress resulting in a clear segregation of expression patterns by carbon source (Fig. 3; Supplementary Figure 1).As expected, culturing the toxigenic isolates in YEP medium resulted in low or no expression of several genes involved in aflatoxin and aflatrem biosynthesis (Figs. 2 and 4). Exceptions to this included members of the sugar cluster (e.g. *aflYc* and *aflYd*) aflatoxin transporters (*aflT*), regulators of aflatoxin production (e.g. *aflR* and *VeA*), and the geranylgeranyl pyrophosphate synthetase gene (*atmG*) which functions upstream of aflatrem biosynthesis in the terpenoid biosynthetic pathway^16^,^34^,^38^,^39^. The expression of the kojic acid SVOP transporter was higher in YEP than in YES in both the toxigenic and atoxigenic isolates Aflaguard and AF36. The expression of this gene was higher in YES than YEP in K54A (Fig. 2C). With this, the expression of metalloproteinases was down-regulated while siderophore biosynthesis genes were up-regulated in some isolates indicating possible iron deficiencies (Supplementary Data 4). The production of aflatoxin and kojic acid also corresponded to observed expression of their corresponding biosynthetic genes. Aflatoxin was found to be produced by toxigenic isolates only in YES (Fig. 5A) while kojic acid was produced in both YES and YEP (Fig. 5B). Interestingly, kojic acid was produced at a higher level in YEP compared to YES (Fig. 5B), and was found to be further stimulated by the addition of H_2_O_2_ in solid culture medium (Fig. 5C).

### Medium carbon source also affects antioxidant and energy metabolism responses

Culturing toxigenic and atoxigenic isolates in YES and YEP media resulted in the upregulation of transcripts encoding antioxidant enzymes including thioredoxin peroxidase, thioredoxin reductase, and components of glutathione metabolism (Supplementary Data 4). Cytochrome p450 monooxygenase genes were also upregulated in response to increasing stress in both the toxigenic and atoxigenic isolates in YEP. Transcripts encoding products involved in proteolysis and gluconeogenesis were also more prevalent with increasing stress in YEP. Chitin catabolic genes were also found to be up-regulated with increasing stress in YEP, particularly in isolates with less oxidative tolerance including NRRL3357, Aflaguard, AF36, and K54A (Supplementary Data 4). In addition, the upregulation of autophagy prevention genes such as *atg22* were observed in NRRL3357 in YEP medium (Supplementary Data 1 and 4)^40^,^41^. In addition, class 3 lipase expression was generally down-regulated in YEP in response to increasing stress, particularly at the highest examined concentrations of H_2_O_2_ relative to the control (Supplementary Data 4). A similar decrease was also observed in K54A in YES (Supplementary Data 3).

### Expression of upstream regulators of oxidative stress responses and carbon utilization

In addition to annotated pathway components as previously described, we also examined the expression of several upstream regulators of secondary metabolite production and oxidative stress responses described in the literature. The expression of bZIP transcription factors such as *atf21* and *atfA*, which have been shown to regulate aflatoxin production in response to oxidative stress *in vitro* during earlier periods in fungal growth^20^, were not significantly regulated in either YES or YEP media in the toxigenic isolates with increasing stress. They were, however, constitutively expressed at an elevated level in all isolates and treatments (Supplementary Data 5).

Several Cis_2_His_2_ (C_2_H_2_) transcription factors were also found to be expressed in the isolates with some previously uncharacterized members such as AFLA_078920 exhibiting higher expression in YES than YEP (Supplementary Data 5). Additional C_2_H_2_ transcription factors such as *creA* (AFLA_134680 and AFLA_134690) and *brlA* (AFLA_082850 and AFLA_082860) were also stably expressed during stress (Supplementary Data 5). In addition to these transcription factors, a mitogen activated protein kinase kinase kinase (MAPKKK), *bck1*, was found to be upregulated in response to increasing levels of oxidative stress (Supplementary Data 5). This MAPKK has been shown to function in coordination with Mkk2 and MpkA^42^, both of which were stably expressed across all isolates and treatments in the study (Supplementary Data 5).

## Discussion

Previously, we observed that the aflatoxin production capabilities of *A. flavus* isolates were correlated with the levels of H_2_O_2_ stress individual isolates could tolerate or survive^32^. Here, we found a related trend with isolates with higher stress tolerance and aflatoxin production exhibiting fewer significant DEGs than atoxigenic, less tolerant isolates in both YES and YEP (Table 1). This may be due to the developmental stage of the tolerant isolates which after seven days in control cultures would exhibit stationary growth patterns^19^. The case for this is bolstered by the observation that other less tolerant isolates generally exhibited up-regulation in aminobenzoate degradation transcripts. These aminobenzoate compounds have been found to reduce both aflatoxin production and mycelial growth^43^. This may imply that more tolerant isolates may possess more rapid and intense responses to stress than the other isolates, and that the cause of this may be related to fungal development (Table 1). Isolate developmental stage may also explain the lack of significant differences in bZIP transcription factor expression observed in this study (Supplementary Data 5). Previously published experiments have mostly examined these genes’ expression and function following approximately 72 hours of fungal growth^20^.

**Figure 6:**
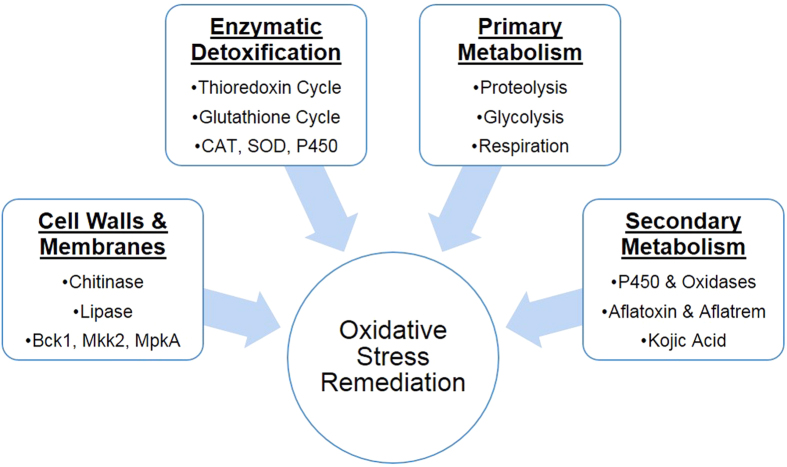
Summary of the overall oxidative stress responses exhibited by *Aspergillus flavus* isolates. The isolates examined in this study exhibited four primary responses to imposed oxidative stress which were influenced by the provided culture medium carbon source (sucrose or peptone). These include: cell wall and cell membrane maintenance and repair through the regulation of chitinases and lipases along with associated signaling pathways; enzymatic detoxification of reactive oxygen species (ROS) through the thioredoxin and glutathione cycles along with enzymes such as catalase (CAT) and superoxide dismutase (SOD); enhancement and maintenance of primary metabolic needs in response to stress; and increased secondary metabolite production to either fix and secrete excess oxygen and ROS, or to limit their formation through co-factor binding or direct antioxidant activity.

In addition to developmental differences, the ability to produce secondary metabolites and antioxidant enzymes, to maintain cell wall integrity, to regulate primary metabolism to meet energetic requirements under stress, and to recycle damaged cellular components were all integral components of the responses of both the toxigenic and atoxigenic isolates (Fig. 6). Cell wall integrity was maintained primarily through the down-regulation of chitinases and chitosanases, although these were found to be up-regulated in YEP under increasing stress indicating a possible reallocation of cellular resources to primary metabolism possibly due to carbon starvation^44^,^45^,^46^. This reallocation of resources may also be due to the limited availability of free glucose and carbon catabolite repression in response to upstream regulation by the C_2_H_2_ transcription factors creA and brlA (Supplementary Data 5) which have been shown to influence the expression of extracellular chitinases in response to carbon starvation^40^,^47^,^48^,^49^,^50^,^51^. In addition, membrane integrity may also be of concern due to the observed regulation of lipase-encoding transcripts in YEP at higher levels of stress (Supplementary Data 4). Their product, a class 3 lipase, breaks down the ester linkages in membrane lipids^51^. A reduction in lipase activity may also reduce the formation of lipoperoxides through the reaction of free fatty acids with ROS^51^.

The catabolism of amino acid components of peptone in the YEP medium would fuel the generation of TCA cycle intermediates such as succinate and fumarate with some being utilized to form acetyl-CoA, the major building block of polyketide and terpenoid mycotoxins such as aflatoxin and aflatrem^16^,^34^. Previous experiments have shown that aflatoxin production was limited or non-existent in YEP, and that supplementation of peptone medium with TCA intermediates would not restore aflatoxin production^52^. However, several monosaccharides such as glucose and fructose have been found to be conducive carbon sources for aflatoxin production^31^. This allows for the possibility that the lack of aflatoxin production observed in YEP (Fig. 5A) may be due to limitations in acetyl or malonyl-CoA availably because of their being directed into primary energy production rather than mycotoxin biosynthesis. Further experimentation will be required to confirm this hypothesis.

In the conducive YES medium, aflatoxin production has been shown to be stimulated by increasing oxidative stress (Fig. 5). Previous studies have also shown that oxidative stress also plays a role as it is required for the initiation of aflatoxin production in *A. flavus* and related species such as *A. parasiticus*^17^. Given the close association of increasing levels of ROS and antioxidant enzyme expression with aflatoxin production, several previous studies have proposed that the production of aflatoxin may function as a supplemental source of antioxidant protection for *A. flavus* and other Aspergilli against oxidative stress^21^,^27^. Increased oxygen consumption and subsequent production of ROS during aflatoxin production in toxigenic isolates has also been demonstrated along with the localization of secondary ROS production to aflatoxisomes inside the fungal cells^26^,^28^. In a recent study, Roze *et al*.^28^ found that this burst in ROS production likely from cytochrome p450 monooxygenase and oxidase activities in aflatoxin biosynthesis may contribute to enhancing oxidative tolerance in conidia. The prevalence of cytochrome p450 genes in both aflatoxin and aflatrem biosynthesis suggests that it is possible similar oxygen consumption and ROS production occurs in both systems (Fig. 4)^34^,^53^. It is also possible that this secondary burst of ROS production may prime expression of antioxidant genes, and that these cytochrome p450 monooxygenase and oxidase enzymes may consume excess oxygen produced by detoxifying enzymes (e.g. CAT, SOD, thioredoxin, etc.) while breaking down ROS, fixing it into aflatoxin and aflatrem which are then secreted from the fungal cells. Therefore, aflatrem biosynthesis may function to enhance oxidative stress tolerance. Further study will be required to better understand the precise biochemical role of aflatoxin and aflatrem in the biology of *A. flavus* and its interactions with host organisms.

In addition to aflatoxin and aflatrem, kojic acid biosynthetic transcripts were also differentially expressed. Kojic acid has been previously described as an antioxidant and is thought to chelate free Fe cations to limit non-enzymatic ROS formation (e.g. Fenton reactions) or through direct reactions with ROS^54^,^55^. Excessive production of kojic acid may result in possible iron deficiencies which may be occurring here given the regulation of both the bck1/mkk2/mpkA signaling pathway and siderophore biosynthetic genes in the isolates (Supplementary Data 4 and 5)^42^,^56^,^57^. With the absence of aflatoxin production in atoxigenic isolates and in YEP, it is possible that kojic acid production may supplement the loss of aflatoxin production-derived stress tolerance. Also, the lack of aflatoxin production and aflatrem biosynthesis gene expression, and the reduced levels of kojic acid produced in solid media assays (Fig. 5C) may partially explain the reduced oxidative stress tolerance of the K54A isolate in comparison to the other examined isolates (Table 1).

Multidrug resistance genes and MFS transporters were also expressed both the toxigenic and atoxigenic isolates which function to enhance fungal tolerance to antibiotic compounds^38^,^58^. In the biological control isolates Aflaguard and AF36, several transcripts regulated in response to oxidative stress may function in antibiotic biosynthesis (Supplementary Data 3 and 4). Together, this indicates oxidative stress may stimulate mechanisms used by the isolates in competition with other organisms both in the host plant and in the soil, and highlights the possibility for the use of ROS in the study biological control competitiveness and drug discovery through the expression of cryptic pathways under oxidative stress^59^.

The connection between oxidative stress and *A. flavus* secondary metabolite production also has implications for improving host plant resistance to aflatoxin contamination. Recent studies have shown that drought sensitive maize lines which have been previously demonstrated to be susceptible to aflatoxin contamination accumulate higher levels of ROS in their leaf and kernel tissues^24^,^25^. Given the previously mentioned correlation between drought stress and aflatoxin contamination^13^, it is possible that these ROS or their reactive byproducts (e.g. H_2_O_2_ and peroxidized lipids) may function in signaling between *A. flavus* and susceptible plants resulting in exacerbated aflatoxin production during infection^8^,^21^,^22^,^24^,^25^. By enhancing the resistance of host tissues to oxidative damage through biomarker and drought tolerance selection in breeding programs, it may be possible to enhance plant resistance to pre-harvest aflatoxin contamination^18^ and needs to be addressed further.

## Conclusion

Transcriptomic analyses of toxigenic and atoxigenic isolates of *A. flavus* and their responses to H_2_O_2_-derived oxidative stress in aflatoxin conducive and non-conducive media revealed that aflatoxin and aflatrem biosynthetic gene expression coincides with the up-regulation of monooxygenase genes. This suggests that the fixation of excess oxygen into these mycotoxins prior to their secretion may provide a degree of oxidative tolerance in *A. flavus*. In addition, kojic acid production was also found to correlate with previously observed isolate tolerance to oxidative stress and may convey additional antioxidant protection in the absence of aflatoxin or aflatrem production by iron chelation. Toxigenic and atoxigenic isolates of *A. flavus* were found to employ similar mechanisms in oxidative stress responses with the exception of secondary metabolite production. Overall, the responses of the isolates focused on four major strategies in countering oxidative stress with individual variations that include cell wall maintenance and repair, enzymatic detoxification of ROS, enhancement and maintenance of primary metabolic needs, and secondary metabolite production. Together, these components work together as the basis of coordinated response to oxidative stress. This study also illustrated the potential roles of secondary metabolite production, including aflatoxin, aflatrem, and kojic acid, in antioxidant responses of *A. flavus*, and their connections to primary metabolism in oxidative stress responses.

## Materials and Methods

### Isolate collection

The *A. flavus* isolates used in the present study were collected from the following sources. The NRRL3357 isolate was obtained from the Northern Regional Research Center, USDA-ARS, Peoria, IL, USA. The AF13, AF36 (NRRL18543), Aflaguard (NRRL21882), and Tox4 isolates were obtained from Dr. Kenneth Damann, Department of Plant Pathology and Crop Physiology, Louisiana State University, Baton Rouge, LA, USA. The K54A isolate was obtained from the Biological Control of Pests Research Unit, USDA-ARS, Stoneville, MS, USA. NRRL3357, AF13, and Tox4 are aflatoxigenic. Aflaguard and AF36 are atoxigenic isolates which possess a complete aflatoxin biosynthetic pathway deletion and a point mutation in the polyketide synthase gene pksA, respectively^36^. The K54A isolate is atoxigenic isolate which has yet to be characterized. The collected isolates were received on potato dextrose agar (PDA) and upon receipt were maintained on V8 agar (20% V8, 1% CaCO_3_, 3% agar).

### Isolate culture conditions

Prior to the experiment, the isolates were grown on V8 agar at 32 °C for 5 days. Fresh conidia were then harvested in 0.1% (v/v) Tween 20 buffer, and this conidial suspension (~4.0 × 10^6^ conidia/mL) was immediately used for inoculation of liquid cultures. Two liquid media were used in the study, an aflatoxin production conducive yeast extract sucrose (YES; 2% yeast extract, 1% sucrose) medium and an aflatoxin production non-conducive yeast extract peptone (YEP; 2% yeast extract, 1% peptone) medium^31^. The concentrations of the media components were optimized based on the findings of Davis *et al*.^60^ and our previous studies^32^.

Prior to inoculation, the media were supplemented with different levels of hydrogen peroxide (H_2_O_2_, 3% stabilized solution) to induce moderate and more severe oxidative stress on the isolates. The concentrations were specific to the individual isolates and were based on our previous findings on the oxidative tolerance (growth inhibiting concentration of H_2_O_2_) of the isolates^32^ as follows. NRRL3357 and Aflaguard were cultured in media supplemented with 0, 10, and 20 mM H_2_O_2_. AF13 and Tox4 were cultured in media supplemented with 0, 10, and 25 mM H_2_O_2_. AF36 and K54A cultured in media supplemented with 0 and 10 mM H_2_O_2_. For each culture, 50 mL of H_2_O_2_ supplemented medium was transferred to a sterile 125 mL Erlenmeyer flask, inoculated with 100 μL conidial suspension, and plugged with a sterile cotton ball. The isolates were then incubated in the dark at 32 °C for 7 days under stationary conditions. This experiment was performed twice and considered as two biological replicates.

### RNA extraction

Following incubation, mycelia were harvested from the flasks using a sterile spatula and immediately flash frozen in liquid nitrogen. The mycelia were then homogenized to a powder using a chilled mortar and pestle and stored at −80 °C until used in RNA extraction. Total RNA was then extracted using an RNeasy Plant Mini Kit with an on-column DNase digestion included during extraction according to the manufacturer’s instructions (Qiagen, Hilden, Germany). The total RNA was quantified using a Nano-Drop ND1000 spectrophotometer (Thermo Scientific, Wilmington, DE, USA), and the quality of the RNA was determined by measuring the RNA integrity numbers (RINs) for each sample using an Agilent 2100 Bioanalyzer (Agilent, Santa Clara, CA, USA). Samples with RIN ≥ 5 were used for library preparation and RNA sequencing.

### Library construction and Illumina sequencing

For library construction, 1 μg of total RNA was used for each sample with biological replicates processed separately for later statistical comparison. Altogether, 32 libraries with 2 biological repeats (total of 64 libraries) were prepared for whole transcriptome sequencing using an Illumina TruSeq RNA Sequencing Kit according to the manufacturer’s instructions (Illumina, San Diego, CA, USA). The prepared libraries were quantified using a Qubit 2.0 fluorometer (Thermo Scientific), and validated using an Agilent 2100 Bioanalyzer (Agilent). Cluster generation for these libraries was done using a cBot (Illumina), followed by 125 bp paired-end sequencing using a HiSeq 2500 platform (Illumina).

### Bioinformatic analysis

Raw sequencing reads obtained for each sample were checked for quality using FastQC v0.11.2 (http://www.bioinformatics.bbsrc.ac.uk/projects/fastqc). The data was then filtered for low quality reads (<Q20) and adapter sequences were removed using Trimmomatic v0.32^61^. The remaining reads were then checked for rRNA contamination by aligning the reads to the SILVA database. Finally, using a Tuxedo protocol, the filtered, quality reads were aligned to the *A. flavus* NRRL3357 genome (GCF_000006275.2) using tophat2 v2.0.13 and bowtie2 v2.2.4^62^,^63^. The filtered reads were assembled by Cufflinks v2.2.1 using the RABT method to estimate their abundance^64^,^65^. The relative expression between isolate-treatment combinations was calculated in terms of Fragments Per Kilobase of exon per Million fragments mapped (FPKM) using the cuffdiff program. A gene was said to be significantly differentially expressed when |log_2_(fold change)| ≥ 2 with an adjusted p-value ≤ 0.05. The assembled transcripts were annotated against NCBI’s non-redundant (nr) protein database using standalone blast-2.2.30+ followed by Gene Ontology (GO) and KEGG pathway analysis using Blast2GO.

### Aflatoxin and kojic acid production assays

In order to validate the induction of kojic acid biosynthesis by increasing levels of H_2_O_2_, a modified method from Bentley^66^ was utilized. Briefly, *A. flavus* isolates were cultured on potato dextrose agar (PDA), PDA amended with 1 mM FeCl_3_, and PDA amended with 1 mM FeCl_3_ along with 15 mM H_2_O_2_. The presence of a bright orange coloration in the medium indicates the chelation of iron by kojic acid allowing for a qualitative comparison of kojic acid biosynthesis between the isolates.

The production of aflatoxin and kojic acid were also evaluated by thin layer chromatography according to methods used by Saruno *et al*.^67^. Briefly, liquid culture medium was collected while harvesting mycelia for RNA isolation, spotted onto a silica gel 20 thin layer chromatography (TLC) plate, and dried at room temperature. The plate was then developed in butanol-acetic acid-water (4:1:5) and dried with warm air. Aflatoxin and kojic acid present within the culture medium samples were then visualized by UV fluorescence with excitation wavelengths of 365 and 254 nm, respectively, and photographed using a Nikon Coolpix L110 digital camera (Nikon, Tokyo, Japan).

## Additional Information

**How to cite this article**: Fountain, J. C. *et al*. Oxidative stress and carbon metabolism influence *Aspergillus flavus* transcriptome composition and secondary metabolite production. *Sci. Rep.* **6**, 38747; doi: 10.1038/srep38747 (2016).

**Publisher's note:** Springer Nature remains neutral with regard to jurisdictional claims in published maps and institutional affiliations.

## Supplementary Material

Supplementary Data 1

Supplementary Data 2

Supplementary Data 3

Supplementary Data 4

Supplementary Data 5

Supplementary Information

## Figures and Tables

**Table 1:** Numbers of significantly differentially expressed genes (DEGs) in *Aspergillus flavus* isolates in YES (yeast extract sucrose) and YEP (yeast extract peptone) media in response to increasing oxidative stress.

Isolate	Toxin*	H_2_O_2_*	YES Medium				YEP Medium	
			0 v 10 mM	0 v 20/25 mM	10 v 20/25 mM	0 v 10 mM	0 v 20/25 mM	10 v 20/25 mM
Tox4	+++	40	4	29	57	19	78	44
AF13	+++	35	6	122	85	49	37	87
NRRL3357	+	20	53	117	112	27	446	738
Aflaguard	−−−	20	16	786	856	41	415	407
AF36	−−−	25	67			17		
K54A	−	15	142			224		

Aflatoxin production and maximum stress tolerance of H_2_O_2_ (mM) were previously determined in Fountain *et al*.^32^. Significant DEGs were identified using Cuffdiff by comparing oxidative stress treatments within each medium with α = 0.05. *Previously observed aflatoxin production and H_2_O_2_ tolerance in Fountain *et al*.^32^. Toxin Production: ‘+++’ High Aflatoxin Producer; ‘+’ Moderate Aflatoxin Producer; ‘‒‒‒’ Atoxigenic Biological Control Isolate; ‘−’ Atoxigenic Isolate.

## References

[b1] FAOSTAT Available: http://faostat.fao.org/. Accessed on February 12, 2016 (2016)

[b2] Andrade P. D. & Caldas E. D. Aflatoxins in cereals: worldwide occurrence and dietary risk assessment. World Mycotoxin J. 8, 415–431 (2015).

[b3] Torres A. M., Barros G. G., Palacios S. A., Chulze S. N. & Battilani P. Review on pre-and post-harvest management of peanuts to minimize aflatoxin contamination. Food Res. Int. 62, 11–19 (2014).

[b4] Kew M. C. Aflatoxins as a cause of hepatocellular carcinoma. J. Gastrointestin. Liver Dis. 22, 305–310 (2013). 24078988

[b5] Williams J. H. . Human aflatoxicosis in developing countries: a review of toxicology, exposure, potential consequences, and interventions. Am. J. Clin. Nutr. 80, 1106–1122 (2004). 15531656 10.1093/ajcn/80.5.1106

[b6] Diener U. L., Asquith R. L. & Dickens J. W. Aflatoxin and *Aspergillus flavus* in corn. So. Coop. Ser. Bull. 279, 112 (1983).

[b7] Diener U. L. . Epidemiology of aflatoxin formation by Aspergillus flavus. Ann. Rev. Phytopathol. 25, 249–270 (1987).

[b8] Fountain J. C. . Environmental influences on maize-*Aspergillus flavus* interactions and aflatoxin production. Front. Microbiol. 5, 40 (2014). 24550905 10.3389/fmicb.2014.00040 PMC3913990

[b9] Guo B., Chen Z. Y., Lee R. D. & Scully B. T. Drought stress and preharvest aflatoxin contamination in agricultural commodity: genetics, genomics and proteomics. J. Int. Plant Biol. 50, 1281–1291 (2008). 10.1111/j.1744-7909.2008.00739.x 19017115

[b10] Pandey M. K. . Advances in *Arachis* genomics for peanut improvement. Biotech. Adv. 30, 639–651 (2012). 10.1016/j.biotechadv.2011.11.001 22094114

[b11] Williams W. P. Breeding for resistance to aflatoxin accumulation in maize. Mycotoxin Res. 22, 27–32 (2006). 23605498 10.1007/BF02954554

[b12] Holbrook C. C., Guo B. Z., Wilson D. M. & Timper P. The US breeding program to develop peanut with drought tolerance and reduced aflatoxin contamination. Peanut Sci. 36, 50–53 (2009).

[b13] Kebede H., Abbas H. K., Fisher D. K. & Bellaloui N. Relationship between aflatoxin contamination and physiological responses of corn plants under drought and heat stress. Toxins, 4, 1385–1403 (2012). 23202322 10.3390/toxins4111385 PMC3509714

[b14] Amaike S. & Keller N. P. Aspergillus flavus. Ann. Rev. Phytopathol. 49, 107–133 (2011). 21513456 10.1146/annurev-phyto-072910-095221

[b15] Guo B. Z. . Crop stress and aflatoxin contamination: perspectives and prevention strategies. Venkateswarlu B., Shankerk A. K., Shanker C., Makeswari M. (Eds.), Crop Stress and Its Management: Perspectives and Strategies, Springer, New York, pp. 399–427 (2012).

[b16] Yu J. . Clustered pathway genes in aflatoxin biosynthesis. App. Environ. Microbiol. 70, 1253–1262 (2004). 10.1128/AEM.70.3.1253-1262.2004 PMC368384 15006741

[b17] Jayashree T. & Subramanyam C. Oxidative stress as a prerequisite for aflatoxin production by *Aspergillus parasiticus*. Free Rad. Biol. Med. 29, 981–985 (2000). 11084286 10.1016/s0891-5849(00)00398-1

[b18] Fountain J. C. . Resistance to *Aspergillus flavus* in maize and peanut: Molecular biology, breeding, environmental stress, and future perspectives. Crop J. 3, 229–237 (2015a).

[b19] Reverberi M. . Modulation of antioxidant defense in *Aspergillus parasiticus* is involved in aflatoxin biosynthesis: a role for the ApyapA gene. Eukaryot. Cell 7, 988–1000 (2008). 18441122 10.1128/EC.00228-07 PMC2446656

[b20] Roze L. V., Chanda A., Wee J., Awad D. & Linz J. E. Stress-related transcription factor AtfB integrates secondary metabolism with oxidative stress response in aspergilli. J. Biol. Chem. 286, 35137–35148 (2011). 21808056 10.1074/jbc.M111.253468 PMC3186425

[b21] Roze L. V., Hong S. Y. & Linz J. E. Aflatoxin biosynthesis: current frontiers. Annu. Rev. Food Sci. Tech. 4, 293–311 (2013). 10.1146/annurev-food-083012-123702 23244396

[b22] Gao X. . Inactivation of the lipoxygenase ZmLOX3 increases susceptibility of maize to *Aspergillus spp*. Mol. Plant Mic. Interact. 22, 222–231 (2009). 10.1094/MPMI-22-2-0222 PMC4545248 19132874

[b23] Howe G. A. & Schilmiller A. L. Oxylipin metabolism in response to stress. Curr. Op. Plant Biol. 5, 230–236 (2002). 10.1016/s1369-5266(02)00250-9 11960741

[b24] Yang L. . Differential accumulation of reactive oxygen and nitrogen species in maize lines with contrasting drought tolerance and aflatoxin resistance. Phytopathology, in press (2016). 10.3390/ijms161024791 PMC4632777 26492235

[b25] Yang L. . Stress sensitivity is associated with differential accumulation of reactive oxygen and nitrogen species in maize genotypes with contrasting levels of drought tolerance. Int. J. Mol. Sci. 16, 24791–24819 (2015). 26492235 10.3390/ijms161024791 PMC4632777

[b26] Narasaiah K. V., Sashidhar R. B. & Subramanyam C. Biochemical analysis of oxidative stress in the production of aflatoxin and its precursor intermediates. Mycopathologia 162, 179–189 (2006). 16944285 10.1007/s11046-006-0052-7

[b27] Reverberi M., Ricelli A., Zjalic S., Fabbri A. A. & Fanelli C. Natural functions of mycotoxins and control of their biosynthesis in fungi. App. Microbiol. Biotech. 87, 899–911 (2010). 10.1007/s00253-010-2657-5 20495914

[b28] Roze L. V. . Aflatoxin biosynthesis is a novel source of reactive oxygen species – A potential redox signal to initiate resistance to oxidative stress? Toxins 7, 1411–1430 (2015). 25928133 10.3390/toxins7051411 PMC4448155

[b29] Davis N. D. & Diener U. L. Growth and aflatoxin production by *Aspergillus parasiticus* from various carbon sources. App. Microbiol. 16, 158 (1968). 10.1128/am.16.1.158-159.1968 PMC547343 5636458

[b30] Yu J., Chang P. K., Bhatnagar D. & Cleveland T. E. Cloning of a sugar utilization gene cluster in *Aspergillus parasiticus. BB* A Gene Struct. Exp. 1493, 211–214 (2000). 10.1016/s0167-4781(00)00148-2 10978525

[b31] Abdollahi A. & Buchanan R. L. Regulation of aflatoxin biosynthesis: induction of aflatoxin production by various carbohydrates. J. Food Sci. 46, 633–635 (1981).

[b32] Fountain J. C. . Effects of hydrogen peroxide on different toxigenic and atoxigenic isolates of *Aspergillus flavus*. Toxins 7, 2985–2999 (2015b). 26251922 10.3390/toxins7082985 PMC4549735

[b33] Valdes J. J., Cameron J. E. & Cole R. J. Aflatrem: a tremorgenic mycotoxin with acute neurotoxic effects. Environ. Health Perspect. 62, 459 (1985). 2867895 10.1289/ehp.8562459 PMC1568710

[b34] Nicholson M. J. . Identification of two aflatrem biosynthesis gene loci in *Aspergillus flavus* and metabolic engineering of *Penicillium paxilli* to elucidate their function. Appl. Environ. Microbiol. 75, 7469–7481 (2009). 19801473 10.1128/AEM.02146-08 PMC2786402

[b35] Terabayashi Y. . Identification and characterization of genes responsible for biosynthesis of kojic acid, an industrially important compound from *Aspergillus oryzae*. Fungal Genet. Biol. 47, 953–961 (2010). 20849972 10.1016/j.fgb.2010.08.014

[b36] Chang P. K. . Identification of genetic defects in the atoxigenic biocontrol strain *Aspergillus flavus* K49 reveals the presence of a competitive recombinant group in field populations. Int. J. Food Microbiol. 154, 192–196 (2012). 22285533 10.1016/j.ijfoodmicro.2012.01.005

[b37] Chang P. K., Horn B. W. & Dorner J. W. Sequence breakpoints in the aflatoxin biosynthesis gene cluster and flanking regions in nonaflatoxigenic *Aspergillus flavus* isolates. Fungal Genet. Biol. 42, 914–923 (2005). 16154781 10.1016/j.fgb.2005.07.004

[b38] Chang P. K., Yu J. & Yu J. H. aflT, a MFS transporter-encoding gene located in the aflatoxin gene cluster, does not have a significant role in aflatoxin secretion. Fungal Genet. Biol. 41, 911–920 (2004). 15341913 10.1016/j.fgb.2004.06.007

[b39] Yu J. H. & Keller N. Regulation of secondary metabolism in filamentous fungi. Annu. Rev. Phytopathol. 43, 437–458 (2005). 16078891 10.1146/annurev.phyto.43.040204.140214

[b40] Yamazaki H. . A chitinase gene, chiB, involved in the autolytic process of *Aspergillus nidulans*. Curr. Genet. 51, 89–98 (2007). 17119968 10.1007/s00294-006-0109-7

[b41] Yang Z., Huang J., Geng J., Nair U. & Klionsky D. J. Atg22 recycles amino acids to link the degradative and recycling functions of autophagy. Mol. Biol. Cell 17, 5094–5104 (2006). 17021250 10.1091/mbc.E06-06-0479 PMC1679675

[b42] Jain R. . The MAP kinase MpkA controls cell wall integrity, oxidative stress response, gliotoxin production and iron adaptation in *Aspergillus fumigatus*. Mol. Microbiol. 82, 39–53 (2011). 21883519 10.1111/j.1365-2958.2011.07778.x PMC3229709

[b43] Chipley J. R. & Uraih N. Inhibition of Aspergillus growth and aflatoxin release by derivatives of benzoic acid. App. Environ. Microbiol. 40, 352–357 (1980). 10.1128/aem.40.2.352-357.1980 PMC291580 6781406

[b44] Emri T., Molnár Z. & Pócsi I. The appearances of autolytic and apoptotic markers are concomitant but differently regulated in carbon-starving *Aspergillus nidulans* cultures. FEMS Microbiol. Letters 251, 297–303 (2005). 10.1016/j.femsle.2005.08.015 16165325

[b45] Nitsche B. M., Jørgensen T. R., Akeroyd M., Meyer V. & Ram A. F. The carbon starvation response of *Aspergillus niger* during submerged cultivation: Insights from the transcriptome and secretome. BMC Genomics 13, 1 (2012). 22873931 10.1186/1471-2164-13-380 PMC3527191

[b46] Emri T., Molnár Z., Szilágyi M. & Pócsi I. Regulation of autolysis in *Aspergillus nidulans*. App. Biochem. Biotech. 151, 211–220 (2008). 10.1007/s12010-008-8174-7 18975147

[b47] Mogensen J., Nielsen H. B., Hofmann G. & Nielsen J. Transcription analysis using high-density micro-arrays of *Aspergillus nidulans* wild-type and creA mutant during growth on glucose or ethanol. Fungal Genet. Biol. 43, 593–603 (2006). 16698295 10.1016/j.fgb.2006.03.003

[b48] Pócsi I. . Asexual sporulation signalling regulates autolysis of *Aspergillus nidulans* via modulating the chitinase ChiB production. J. App. Microbiol. 107, 514–523 (2009). 10.1111/j.1365-2672.2009.04237.x 19486415

[b49] Roy P., Lockington R. A. & Kelly J. M. CreA-mediated repression in *Aspergillus nidulans* does not require transcriptional auto-regulation, regulated intracellular localisation or degradation of CreA. Fungal Genet. Biol. 45, 657–670 (2008). 18063396 10.1016/j.fgb.2007.10.016

[b50] Strauss J. . The function of CreA, the carbon catabolite repressor of *Aspergillus nidulans*, is regulated at the transcriptional and post‐transcriptional level. Mol. Microbiol. 32, 169–178 (1999). 10216870 10.1046/j.1365-2958.1999.01341.x

[b51] Bhattacharjee S. Reactive oxygen species and oxidative burst: roles in stress, senescence and signal. Curr. Sci. India. 89, 1113–1121 (2005).

[b52] Yan S., Liang Y., Zhang J. & Liu C. M. *Aspergillus flavus* grown in peptone as the carbon source exhibits spore density-and peptone concentration-dependent aflatoxin biosynthesis. BMC Microbiol. 12, 106 (2012). 22694821 10.1186/1471-2180-12-106 PMC3412747

[b53] Duran R. M., Cary J. W. & Calvo A. M. Production of cyclopiazonic acid, aflatrem, and aflatoxin by *Aspergillus flavus* is regulated by veA, a gene necessary for sclerotial formation. App. Microbiol. Biotech. 73, 1158–1168 (2007). 10.1007/s00253-006-0581-5 16988822

[b54] Chang P. K. . Loss of msnA, a putative stress regulatory gene, in *Aspergillus parasiticus* and *Aspergillus flavus* increased production of conidia, aflatoxins and kojic acid. Toxins 3, 82–104 (2011). 22069691 10.3390/toxins3010082 PMC3210457

[b55] Fenton H. J. H. Oxidation of tartaric acid in presence of iron. J. Chem. Soc. Trans. 65, 899–910 (1894).

[b56] Valiante V., Heinekamp T., Jain R., Härtl A. & Brakhage A. A. The mitogen-activated protein kinase MpkA of *Aspergillus fumigatus* regulates cell wall signaling and oxidative stress response. Fungal Genet. Biol. 45, 618–627 (2008). 17981060 10.1016/j.fgb.2007.09.006

[b57] Valiante V., Jain R., Heinekamp T. & Brakhage A. A. The MpkA MAP kinase module regulates cell wall integrity signaling and pyomelanin formation in *Aspergillus fumigatus*. Fungal Genet. Biol. 46, 909–918 (2009). 19715768 10.1016/j.fgb.2009.08.005

[b58] Tobin M. B., Peery R. B. & Skatrud P. L. Genes encoding multiple drug resistance-like proteins in *Aspergillus fumigatus* and *Aspergillus flavus*. Gene 200, 11–23 (1997). 9373135 10.1016/s0378-1119(97)00281-3

[b59] Brakhage A. A. & Schroeckh V. Fungal secondary metabolites–strategies to activate silent gene clusters. Fungal Genet. Biol. 48, 15–22 (2011). 20433937 10.1016/j.fgb.2010.04.004

[b60] Davis N. D., Diener U. L. & Agnihotri V. P. Production of aflatoxins B1 and G1 in chemically defined medium. Mycopathol. Mycol. App. 31, 251–256 (1967). 10.1007/BF02053422 6031300

[b61] Bolger A., Lohse M. & Usadel B. Trimmomatic: a flexible trimmer for Illumina sequence data. Bioinformatics. 30, 1–7 (2014). 24695404 10.1093/bioinformatics/btu170 PMC4103590

[b62] Kim D. . TopHat2: accurate alignment of transcriptomes in the presence of insertions, deletions and gene fusions. Genome Biol. 14, R36 (2013). 23618408 10.1186/gb-2013-14-4-r36 PMC4053844

[b63] Langmead B., Trapnell C., Pop M. & Salzberg S. Ultrafast and memory-efficient alignment of short DNA sequences to the human genome. Genome Biol. 10, R25 (2009). 19261174 10.1186/gb-2009-10-3-r25 PMC2690996

[b64] Roberts A., Pimentel H., Trapnell C. & Pachter L. Identification of novel transcripts in annotated genomes using RNA-Seq. Bioinformatics. 27, 2325–2329 (2011). 21697122 10.1093/bioinformatics/btr355

[b65] Trapnell C. . Differential analysis of gene regulation at transcript resolution with RNA-seq. Nature Biotechnol. 31, 46–54 (2013). 23222703 10.1038/nbt.2450 PMC3869392

[b66] Bentley R. Preparation and analysis of kojic acid. Methods in Enzymol. 3, 238–241 (1957).

[b67] Saruno R., Kato F. & Ikeno T. Kojic acid, a tyrosinase inhibitor from *Aspergillus albus*. Ag. Biol. Chem. 43, 1337–1338 (1979).

