# Supplementary material for: Oxidative stress and carbon metabolism influence Aspergillus flavus transcriptome composition and secondary metabolite production
Source: Sci Rep. 2016 Dec 12;6:38747. doi: 10.1038/srep38747 (PMC5150527; doi:10.1038/srep38747)
Supplement: Supplementary Information [file srep38747-s6.pdf]

# Supplementary Information:

# Oxidative stress and carbon metabolism influence *Aspergillus flavus* transcriptome composition and secondary metabolite production

Jake C. Fountain[1,2,†], Prasad Bajaj[3,†], Manish Pandey[3], Spurthi N. Nayak[3], Liming Yang[1,2], Vinay Kumar[3], Ashwin S. Jayale[3], Anu Chitikineni[3], Weijian Zhuang[4], Brian T. Scully[5], R. Dewey Lee[6], Robert C. Kemerait[1], Rajeev K. Varshney[3,*], Baozhu Guo[2,*]

[1]Department of Plant Pathology, University of Georgia, Tifton, GA, USA

[2]USDA-ARS Crop Protection and Management Research Unit, Tifton, GA, USA

[3]International Crop Research Institute for the Semi-Arid Tropics (ICRISAT), Hyderabad, Telangana, India

[4]Fujian Agricultural and Forestry University, Fuzhou, Fujian, China

[5]USDA-ARS US Horticultural Research Laboratory, Fort Pierce, FL, USA

[6]Department of Crop and Soil Sciences, University of Georgia, Tifton, GA, USA

[†]Equal contribution.

Author e-mail addresses: J.C. Fountain: jfount1@uga.edu; P. Bajaj: p. bajaj@cgiar.org; M. Pandey: m.pandey@cgiar.org; S.N. Nayak: s.nayak@cgiar.org; V. Kumar: vinay.kumar@cgiar.org; A.S. Jayale: ashu8386@gmail.com; A. Chitikineni: a.chitikineni@cgiar.org; L. Yang: yanglm@uga.edu; W. Zhuang: weijianz1@163.com; B. Sully: brian.scully@ars.usda.gov; R.D. Lee: deweylee@uga.edu; R.C. Kemerait: kemerait@uga.edu; R.K. Varshney: r.k.varshney@cgiar.org; B. Guo: baozhu.guo@ars.usda.gov

***Corresponding Authors:**
Dr. Baozhu Guo
baozhu.guo@ars.usda.gov

Dr. Rajeev K. Varshney
r.k.varshney@cgiar.org

## Supplemental Figure Legend

**Supplementary Figure 1. Dendrogram analysis of isolate gene expression profiles under increasing oxidative stress with different carbon sources.** Dendrogram analysis of isolate expression profiles shows a clear segregation of the isolates into separate clades based on medium carbon source with the first and second clades comprised mostly of YES (sucrose) and YEP (peptone) cultured isolates, respectively. A third clade separated and contains NRRL3357, Aflaguard, and K54A with high levels of stress. Within each clade, toxigenic and atoxigenic isolates tend to cluster separately with the highly toxigenic or biological control isolates clustering separately from NRRL3357 and K54A, respectively.

**Supplementary Figure 1**

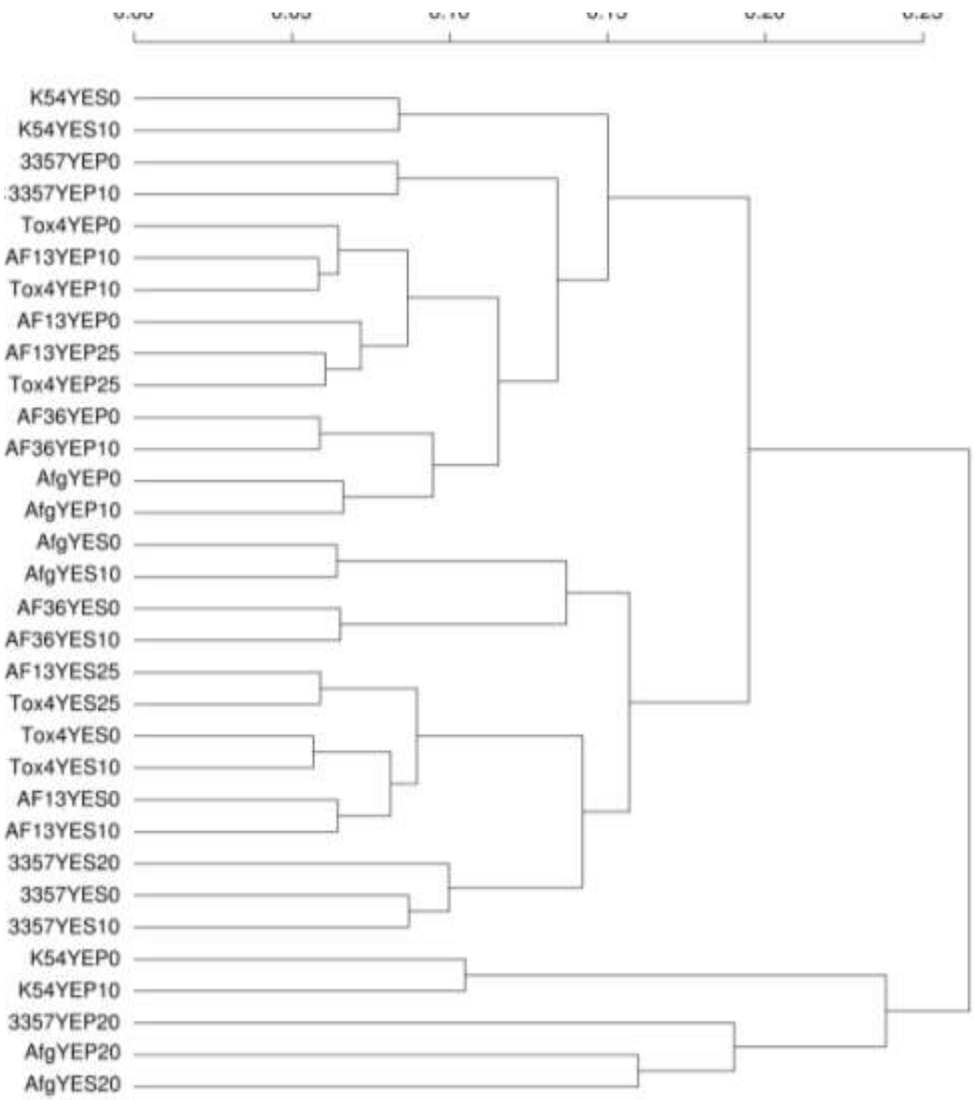

## Supplemental Tables

**Supplementary Table 1.** Summary of transcriptome data.

| | |
|---|---|
| Total Raw Data (bp) | 588,219,207,500 |
| Total Raw Reads | 4,705,753,660 |
| Avg. Raw Reads per Sample | 73,527,401 |
| Total Filtered Data (bp) | 282,573,713,964 |
| Total Filtered Reads | 2,531,887,207 |
| Avg. Filtered Reads per Sample | 40,836,890 |
| Min. Filtered Reads per Sample | 29,013,626 |
| Max. Filtered Reads per Sample | 69,024,854 |
| Avg. GC Content (%) | 51.71 |
| Avg. Reference Mapping (%) | 92.27 |

**Supplementary Table 2.** Gene expression FPKM data for aflatoxin, aflatrem, and kojic acid biosynthesis genes.

| Tox4YES0 | Tox4YES10 | Tox4YES25 | Tox4YEP0 | Tox4YEP10 | Tox4YEP25 | AF13YES0 | AF13YES10 | AF13YES25 | AF13YEP0 | AF13YEP10 | AF13YEP25 | 3357YES0 | 3357YES10 | 3357YES20 | 3357YEP0 | 3357YEP10 | 3357YEP20 | AF36YES0 | AF36YES10 | AF36YEP0 | AF36YEP10 | AfgYES0 | AfgYES10 | AfgYES20 | AfgYEP0 | AfgYEP10 | AfgYEP20 | K54AYES0 | K54AYES10 | K54AYEP0 | K54AYEP10 | Gene | |
|---|---|---|---|---|---|---|---|---|---|---|---|---|---|---|---|---|---|---|---|---|---|---|---|---|---|---|---|---|---|---|---|---|---|
| 11 | 10 | 8 | 10 | 8 | 7 | 9 | 10 | 11 | 9 | 8 | 8 | 10 | 12 | 15 | 8 | 5 | 6 | 11 | 10 | 8 | 7 | 11 | 10 | 7 | 11 | 8 | 4 | 7 | 9 | 12 | 6 | aflA / fas-2 | Aflatoxin |
| 6 | 6 | 6 | 2 | 2 | 2 | 4 | 4 | 8 | 2 | 2 | 2 | 6 | 9 | 14 | 1 | 1 | 3 | 3 | 2 | 1 | 1 | 6 | 5 | 3 | 2 | 2 | 1 | 1 | 2 | 4 | 1 | aflB / fas-1 | |
| 3 | 3 | 3 | 2 | 2 | 2 | 3 | 3 | 5 | 2 | 2 | 2 | 4 | 8 | 21 | 2 | 1 | 4 | 1 | 1 | 1 | 0 | 1 | 1 | 2 | 1 | 1 | 0 | 2 | 2 | 11 | 1 | aflC / pksA | |
| 3 | 2 | 2 | 2 | 2 | 1 | 4 | 3 | 7 | 1 | 2 | 3 | 4 | 15 | 24 | 2 | 1 | 2 | 1 | 1 | 1 | 0 | 1 | 1 | 7 | 1 | 1 | 0 | 2 | 2 | 5 | 1 | aflCa / hypC | |
| 7 | 6 | 6 | 4 | 2 | 3 | 5 | 5 | 16 | 3 | 3 | 4 | 8 | 21 | 41 | 3 | 4 | 6 | 8 | 7 | 5 | 5 | 8 | 6 | 12 | 4 | 5 | 4 | 12 | 10 | 15 | 7 | aflD / nor-1 | |
| 47 | 47 | 64 | 6 | 13 | 21 | 42 | 46 | 179 | 11 | 13 | 18 | 53 | 135 | 163 | 13 | 10 | 22 | 27 | 21 | 27 | 39 | 112 | 72 | 149 | 36 | 41 | 34 | 11 | 24 | 3 | 6 | aflE/ norA | |
| 11 | 9 | 9 | 3 | 3 | 3 | 9 | 9 | 9 | 4 | 3 | 3 | 9 | 10 | 8 | 5 | 3 | 0 | 4 | 4 | 2 | 2 | 5 | 5 | 1 | 2 | 1 | 1 | 9 | 7 | 1 | 1 | aflF / norB | |
| 0 | 0 | 0 | 0 | 0 | 0 | 0 | 0 | 5 | 0 | 0 | 0 | 0 | 5 | 9 | 0 | 0 | 0 | 0 | 0 | 0 | 0 | 0 | 0 | 3 | 0 | 0 | 0 | 0 | 0 | 0 | 0 | aflG/ avnA | |
| 0 | 0 | 0 | 0 | 0 | 0 | 0 | 0 | 1 | 0 | 0 | 0 | 1 | 13 | 25 | 0 | 0 | 0 | 0 | 0 | 0 | 0 | 0 | 0 | 1 | 0 | 0 | 0 | 0 | 0 | 0 | 0 | aflI/ avfA | |
| 0 | 0 | 0 | 0 | 0 | 0 | 0 | 0 | 4 | 0 | 0 | 0 | 1 | 11 | 25 | 0 | 0 | 0 | 0 | 0 | 0 | 0 | 0 | 0 | 6 | 0 | 0 | 0 | 0 | 0 | 0 | 0 | aflK/ vbs | |
| 0 | 0 | 0 | 0 | 0 | 0 | 0 | 0 | 3 | 0 | 0 | 0 | 1 | 17 | 40 | 0 | 0 | 0 | 0 | 0 | 0 | 0 | 0 | 0 | 1 | 0 | 0 | 0 | 0 | 0 | 0 | 0 | aflM/ ver-1 | |
| 0 | 0 | 0 | 0 | 0 | 0 | 0 | 0 | 0 | 0 | 0 | 0 | 1 | 8 | 17 | 0 | 0 | 0 | 0 | 0 | 0 | 0 | 0 | 0 | 0 | 0 | 0 | 0 | 0 | 0 | 0 | 0 | aflMa/ hypE | |
| 107 | 101 | 102 | 136 | 153 | 137 | 111 | 114 | 195 | 199 | 163 | 157 | 110 | 225 | 217 | 199 | 215 | 11 | 135 | 100 | 229 | 235 | 162 | 164 | 122 | 189 | 182 | 184 | 194 | 159 | 280 | 379 | aflNa/ hypD | |
| 0 | 0 | 0 | 0 | 0 | 0 | 0 | 0 | 0 | 0 | 0 | 0 | 1 | 13 | 28 | 0 | 0 | 0 | 0 | 0 | 0 | 0 | 0 | 0 | 0 | 0 | 0 | 0 | 0 | 0 | 0 | 0 | aflO/ omtB | |
| 0 | 0 | 0 | 0 | 0 | 0 | 0 | 0 | 2 | 0 | 0 | 0 | 1 | 8 | 19 | 0 | 0 | 0 | 0 | 0 | 0 | 0 | 0 | 0 | 4 | 0 | 0 | 0 | 0 | 0 | 0 | 0 | aflP/ omtA | |
| 0 | 0 | 0 | 0 | 0 | 0 | 0 | 0 | 1 | 0 | 0 | 0 | 0 | 2 | 4 | 0 | 0 | 0 | 0 | 0 | 0 | 0 | 0 | 0 | 1 | 0 | 0 | 0 | 0 | 0 | 0 | 0 | aflQ/ ordA | |
| 2 | 2 | 2 | 4 | 2 | 2 | 2 | 2 | 2 | 4 | 3 | 3 | 2 | 2 | 2 | 4 | 2 | 1 | 5 | 6 | 4 | 3 | 3 | 4 | 1 | 4 | 2 | 1 | 2 | 1 | 2 | 1 | aflR-like C6 TF | |
| 97 | 100 | 107 | 16 | 32 | 50 | 93 | 83 | 113 | 32 | 32 | 42 | 86 | 86 | 93 | 33 | 31 | 32 | 44 | 33 | 44 | 54 | 118 | 81 | 65 | 48 | 53 | 51 | 42 | 78 | 5 | 17 | aflR | |
| 17 | 13 | 14 | 18 | 17 | 19 | 11 | 10 | 14 | 20 | 17 | 18 | 14 | 18 | 29 | 22 | 21 | 41 | 39 | 35 | 23 | 18 | 28 | 35 | 27 | 19 | 18 | 19 | 25 | 21 | 267 | 186 | aflT MFS efflux pump | |
| 111 | 119 | 126 | 36 | 42 | 44 | 124 | 123 | 125 | 38 | 44 | 38 | 110 | 96 | 90 | 53 | 37 | 1 | 56 | 68 | 30 | 33 | 77 | 72 | 21 | 38 | 28 | 11 | 72 | 72 | 5 | 5 | aflT transmembrane protein | |
| 0 | 0 | 0 | 0 | 0 | 0 | 0 | 0 | 3 | 0 | 0 | 0 | 0 | 5 | 11 | 0 | 0 | 0 | 0 | 0 | 0 | 0 | 0 | 0 | 3 | 0 | 0 | 0 | 0 | 0 | 0 | 0 | aflV/ cypX | |
| 0 | 0 | 0 | 0 | 0 | 0 | 0 | 0 | 4 | 0 | 0 | 0 | 1 | 6 | 12 | 0 | 0 | 1 | 0 | 0 | 0 | 0 | 0 | 0 | 3 | 0 | 0 | 0 | 0 | 0 | 0 | 0 | aflW/ moxY | |
| 0 | 0 | 0 | 0 | 0 | 0 | 0 | 0 | 6 | 0 | 0 | 0 | 1 | 9 | 13 | 0 | 0 | 5 | 0 | 0 | 0 | 0 | 0 | 0 | 4 | 0 | 0 | 0 | 0 | 0 | 2 | 0 | aflX/ ordB | |
| 0 | 0 | 0 | 0 | 0 | 0 | 0 | 0 | 3 | 0 | 0 | 0 | 0 | 4 | 8 | 0 | 0 | 1 | 0 | 0 | 0 | 0 | 0 | 0 | 2 | 0 | 0 | 0 | 0 | 0 | 2 | 0 | aflY/ hypA | |
| 0 | 0 | 0 | 0 | 0 | 0 | 0 | 0 | 1 | 0 | 0 | 0 | 0 | 1 | 3 | 0 | 0 | 0 | 0 | 0 | 0 | 0 | 0 | 0 | 1 | 0 | 0 | 0 | 0 | 0 | 1 | 0 | aflYa/ nadA | |
| 6 | 7 | 7 | 12 | 11 | 9 | 7 | 9 | 7 | 13 | 13 | 11 | 11 | 8 | 7 | 12 | 6 | 8 | 8 | 8 | 10 | 10 | 8 | 5 | 6 | 12 | 11 | 7 | 9 | 8 | 23 | 14 | aflYb/ hxtA | |
| 6 | 6 | 6 | 10 | 9 | 8 | 6 | 6 | 6 | 9 | 11 | 10 | 8 | 9 | 6 | 11 | 7 | 5 | 8 | 9 | 10 | 10 | 6 | 5 | 7 | 11 | 10 | 7 | 11 | 9 | 15 | 11 | aflYc/ glcA | |
| 5 | 5 | 5 | 6 | 7 | 5 | 5 | 4 | 5 | 6 | 6 | 6 | 7 | 7 | 6 | 11 | 7 | 6 | 7 | 8 | 9 | 7 | 5 | 4 | 7 | 9 | 7 | 4 | 10 | 10 | 13 | 8 | aflYd/ sugR | |
| 25 | 24 | 28 | 18 | 18 | 19 | 32 | 32 | 32 | 19 | 18 | 19 | 24 | 25 | 16 | 22 | 19 | 22 | 13 | 11 | 18 | 18 | 15 | 13 | 25 | 21 | 21 | 29 | 12 | 12 | 9 | 11 | aflYg/ npgA | |
| 33 | 39 | 28 | 28 | 26 | 23 | 40 | 44 | 25 | 21 | 24 | 23 | 62 | 58 | 41 | 23 | 20 | 19 | 38 | 49 | 18 | 18 | 30 | 27 | 22 | 35 | 31 | 21 | 61 | 71 | 32 | 24 | VeA | |
| 179 | 145 | 82 | 0 | 0 | 0 | 51 | 42 | 31 | 0 | 0 | 0 | 9 | 79 | 28 | 0 | 0 | 0 | 201 | 152 | 3 | 9 | 17 | 16 | 3 | 0 | 1 | 0 | 0 | 1 | 0 | 0 | atmB | Aflatrem |
| 134 | 197 | 82 | 0 | 0 | 0 | 73 | 43 | 20 | 0 | 0 | 0 | 1 | 139 | 1 | 0 | 0 | 0 | 221 | 170 | 0 | 1 | 1 | 1 | 0 | 0 | 0 | 0 | 0 | 1 | 0 | 0 | atmC | |
| 62 | 74 | 32 | 0 | 0 | 0 | 40 | 31 | 9 | 0 | 0 | 0 | 5 | 31 | 6 | 0 | 0 | 0 | 183 | 125 | 1 | 1 | 29 | 45 | 0 | 0 | 0 | 0 | 0 | 0 | 0 | 1 | atmD | |
| 9 | 10 | 10 | 5 | 10 | 11 | 20 | 14 | 21 | 3 | 5 | 4 | 36 | 21 | 32 | 3 | 3 | 0 | 7 | 5 | 5 | 5 | 13 | 14 | 10 | 4 | 6 | 5 | 5 | 10 | 0 | 1 | atmG | |
| 58 | 80 | 41 | 0 | 0 | 0 | 29 | 18 | 10 | 0 | 0 | 0 | 1 | 51 | 1 | 0 | 0 | 0 | 102 | 76 | 0 | 1 | 1 | 0 | 0 | 0 | 0 | 0 | 0 | 0 | 0 | 0 | atmM | |
| 175 | 183 | 102 | 0 | 1 | 0 | 75 | 53 | 33 | 0 | 0 | 0 | 13 | 109 | 35 | 0 | 0 | 0 | 255 | 207 | 2 | 5 | 16 | 19 | 3 | 0 | 1 | 1 | 0 | 1 | 0 | 0 | atmP | |
| 214 | 191 | 139 | 1 | 1 | 1 | 97 | 69 | 82 | 0 | 1 | 1 | 49 | 158 | 146 | 0 | 0 | 0 | 332 | 239 | 11 | 20 | 85 | 112 | 13 | 1 | 2 | 3 | 2 | 12 | 0 | 0 | atmQ | |
| 3 | 3 | 3 | 3 | 3 | 2 | 4 | 3 | 3 | 2 | 2 | 2 | 5 | 4 | 4 | 2 | 3 | 26 | 5 | 4 | 4 | 3 | 6 | 5 | 56 | 4 | 3 | 3 | 4 | 4 | 5 | 3 | kojA | Kojic Acid |
| 18 | 20 | 16 | 18 | 14 | 17 | 16 | 16 | 16 | 18 | 16 | 18 | 21 | 26 | 23 | 22 | 19 | 28 | 20 | 16 | 24 | 19 | 20 | 21 | 40 | 21 | 17 | 15 | 38 | 24 | 26 | 20 | kojR/ kojT | |
| 52 | 31 | 2 | 2853 | 674 | 359 | 4 | 2 | 1 | 1721 | 559 | 788 | 15 | 181 | 16 | 2689 | 4726 | 2 | 411 | 160 | 1531 | 421 | 2 | 5 | 1 | 361 | 357 | 3 | 2275 | 540 | 25 | 15 | Synaptic Vesicle Transporter SVOP | |
| 463 | 470 | 189 | 168 | 98 | 42 | 496 | 367 | 69 | 76 | 70 | 37 | 333 | 466 | 106 | 79 | 44 | 5 | 629 | 691 | 38 | 22 | 182 | 164 | 3 | 75 | 73 | 7 | 79 | 75 | 30 | 17 | UNC93-like MFS Transporter | |

## Supplemental Data Files

**Supplementary Data 1.** Gene expression FPKM data for all isolates and treatments.

**Supplementary Data 2.** Genes expressed exclusively in toxigenic or atoxigenic isolates.

**Supplementary Data 3.** Gene ontology (GO) and KEGG pathway analysis for isolates in YES medium.

**Supplementary Data 4.** Gene ontology (GO) and KEGG pathway analysis for isolates in YEP medium.

**Supplementary Data 5.** Gene expression FPKM data for select regulatory transcription factors and kinases.
